# A double-blind randomized controlled trial to assess the effect of bright light therapy on depression in patients with Parkinson’s disease

**DOI:** 10.1186/s12888-016-1050-z

**Published:** 2016-10-21

**Authors:** Sonja Rutten, Chris Vriend, Jan H. Smit, Henk W. Berendse, Adriaan W. Hoogendoorn, Odile A. van den Heuvel, Ysbrand D. van der Werf

**Affiliations:** 1 Department of Psychiatry, VU University Medical Center/GGZ inGeest, A.J. Ernststraat 887, 1081 HL Amsterdam, The Netherlands; 2 Department of Anatomy and Neurosciences, VU University Medical Center, Gustav Mahlerlaan 3004, 1000 MB Amsterdam, The Netherlands; 3 Amsterdam Neuroscience, de Boelelaan 1085, 1081 HV Amsterdam, The Netherlands; 4 Department of Neurology, VU University Medical Center, PO Box 7057, 1007 MB Amsterdam, The Netherlands

**Keywords:** Bright light therapy, Parkinson’s disease, Depressive disorder, Chronotherapy, Neuropsychiatry, Randomized controlled trial

## Abstract

**Background:**

A disturbed circadian rhythm seems to be a causal factor in the occurrence of depressive disorders in patients with Parkinson’s disease (PD). The circadian rhythm can be restored with light. Therefore, Bright Light Therapy (BLT) might be a new treatment option for depression in PD patients.

**Methods/design:**

In this double-blind controlled trial, 84 subjects with idiopathic PD are randomized to either BLT or a control light condition. The BLT condition emits white light with an intensity of 10,000 Lux, while the control device emits dim white light of 200 Lux, which is presumed to be too low to influence the circadian rhythm. Subjects receive 30 min of home treatment twice daily for three months. Timing of treatment is based on the individual chronotype. After finishing treatment, subjects enter a follow-up period of six months. The primary outcome of the study is the severity of depressive symptoms, as measured with the Hamilton Depression Rating Scale. Secondary outcomes are alternative depression measures, objective and subjective sleep measures, and salivary melatonin and cortisol concentrations. For exploratory purposes, we also assess the effects on motor symptoms, global cognitive function, comorbid psychiatric disorders, quality of life and caregiver burden. Data will be analyzed using a linear mixed models analysis.

**Discussion:**

Performing a placebo-controlled trial on the effects of BLT in PD patients is challenging, as the appearance of the light may provide clues on the treatment condition. Moreover, fixed treatment times lead to an improved sleep-wake rhythm, which also influences the circadian system. With our study design, we do not compare BLT to placebo treatment, i.e. an ineffective control treatment. Rather, we compare structuring of the sleep-wake cycle in both conditions with additional BLT in the experimental condition, and additional dim light in the control condition. Participants are not informed about the exact details of the two light devices and the expected therapeutic effect, and expectancies are rated prior to the start of treatment. Ideally, the design of a future study on BLT should include two extra treatment arms where BLT and control light are administered at random times.

**Trial registration:**

This trial was registered on ClinicalTrials.gov on May 17th 2012 (ClinicalTrials.gov Identifier: NCT01604876).

## Background

In patients with Parkinson’s disease (PD), clinically relevant symptoms of depression occur in up to 35 %, with approximately 17 % fulfilling criteria for major depressive disorder (MDD) [1]. Depression has a major impact on overall functioning: depressed PD patients score lower on scales assessing activities of daily living, show a more rapid deterioration of motor and cognitive functioning, and have a higher mortality [2–4]. Depression is highly correlated with insomnia in PD [5–7], both contributing to a reduced quality of life in PD patients and their caregivers [3, 5].

Current treatment options for depression in PD are limited. Antidepressants can have side effects, such as orthostatic hypotension, sedation, and anticholinergic adverse effects, and may aggravate motor symptoms [8–10]. Cognitive behavioral therapy might be effective for the treatment of depression in PD patients, but has not been extensively studied yet [11]. To date, there are no evidence-based treatment options for insomnia in PD, and treatment with hypnotics can have adverse effects [7]. For patients with cognitive dysfunctions, which are common in PD [12], psychotherapy is not a always a feasible treatment option. An alternative treatment for depressive disorders and insomnia in PD patients is therefore needed.

### Involvement of the circadian system in depression in PD

The circadian rhythm consists of neuroendocrine and behavioral cycles of approximately 24 h. This rhythm is generated by the ‘biological clock’, the suprachiasmatic nucleus (SCN), which is located in the hypothalamus [13]. The SCN promotes wakefulness during the day and sleep during the night, providing the body with a pattern of rest and activity that corresponds to the day-night cycle. Moreover, the SCN is involved in mood regulation through the influence on neurotransmitter systems [14].

The circadian system regulates the secretion of several hormones, including melatonin and cortisol [15]. Under normal circumstances, the pineal gland starts secreting melatonin two to three hours prior to bedtime to promote sleep [15]. When plasma or saliva samples are taken under dim light conditions, this rise in melatonin levels is referred to as the dim-light melatonin offset (DLMO) [16]. In the morning, the secretion of melatonin is inhibited by exposure to light, leading to a decrease in melatonin concentrations [15]. Cortisol concentrations, on the other hand, peak in the morning, reflecting the response of the hypothalamic-pituitary-adrenal axis to awakening [15, 17]. This peak is referred to as the cortisol awakening response [18]. A more indirect measure of the circadian rhythm is the chronotype: the propensity of an individual to be active or asleep during a 24-hour period, with ‘morning’ and ‘evening types’ at the extremes of the spectrum. The chronotype can be measured with the Morningness – Eveningness Questionnaire (MEQ) [19]. Previous studies have shown a significant correlation between the MEQ score and the DLMO [19, 20].

Since the endogenous rhythm of the SCN is slightly different from the societal 24-hour day-night cycle, it has to be entrained by input signals from the environment called ‘zeitgebers’, such as light, physical activity and food intake [13, 21]. Disrupting input to the biological clock can lead to a disturbed circadian rhythm, as reflected in a shift or change in amplitude of the cortisol and melatonin secretion patterns. A misalignment of the circadian rhythm is associated with depressive symptoms and insomnia [22–24].

PD patients are prone to desynchronization of the circadian rhythm, due to dopamine deficiency and conflicting input to the SCN [25]. Indeed, a disturbed circadian rhythm is observed in this population. Three studies described a phase advance of melatonin secretion in PD subjects [26–28]. Moreover, a decrease in the amplitude and total amount of melatonin and cortisol secretion has been observed [29, 30]. In another study, the pattern of cortisol secretion was also blunted, but total cortisol concentrations were increased in PD subjects compared to healthy controls [31]. These changes in melatonin and cortisol secretion patterns have also been reported in non-PD subjects with a depressive disorder or insomnia [32–34]. Based on these findings, we hypothesize that a disturbed circadian rhythm is involved in the pathophysiology of depression and insomnia in PD.

### Bright light therapy

Light stimulates melanopsin-containing ganglion cells in the retina, providing the SCN with a ‘daytime’ signal via the retinohypothalamic tract [13]. Bright Light Therapy (BLT) therefore acts as a strong zeitgeber that can restore circadian rhythmicity. Light exposure in the morning advances the circadian rhythm and is effective in the treatment of depressive disorders [35–39], while evening light delays the rhythm and is thought to be more effective in the treatment of early awakening insomnia [32]. Since PD patients have an advanced circadian rhythm [26–28], evening light might be effective in treating not only insomnia, but also depressive symptoms in this population.

In their frequently cited meta-analysis, Golden et al. (2005) concluded that BLT is effective in the treatment of depression in non-PD samples [36]. Recent meta-analyses are more cautious in drawing conclusions on the efficacy of BLT due to limitations of the performed studies, e.g. small and heterogeneous study samples, short treatment duration and lack of a proper placebo condition [35, 37, 38]. The same issue was raised in a Cochrane review on the effects of BLT for treating insomnia in adults aged 60 years and older [40]. However, since treatment effects of pharmacotherapy alone for these disorders are limited, and BLT is a low-cost treatment with a relatively high safety and tolerability [41], it is a treatment option that deserves further research.

In PD, the effects of BLT have been evaluated in two pilot studies [42, 43] and one retrospective, open label study [44]. These studies demonstrated positive effects on depressive symptoms, sleep and motor symptoms. However, depression was not the primary outcome measure in all studies, and all studies had methodological limitations. Therefore, further research on the efficacy of BLT in the treatment of PD patients with a depressive disorder is warranted.

## Methods

This study aims to investigate whether BLT is more effective in reducing depressive symptoms in patients with PD and a depressive disorder, than a control light device. For secondary research purposes, we assess the effects of BLT on insomnia and circadian rhythmicity.

### Study design and procedures

The design of this study is a double-blind randomized controlled clinical trial. An overview of all study procedures is given in Table 1.

**Table 1:**
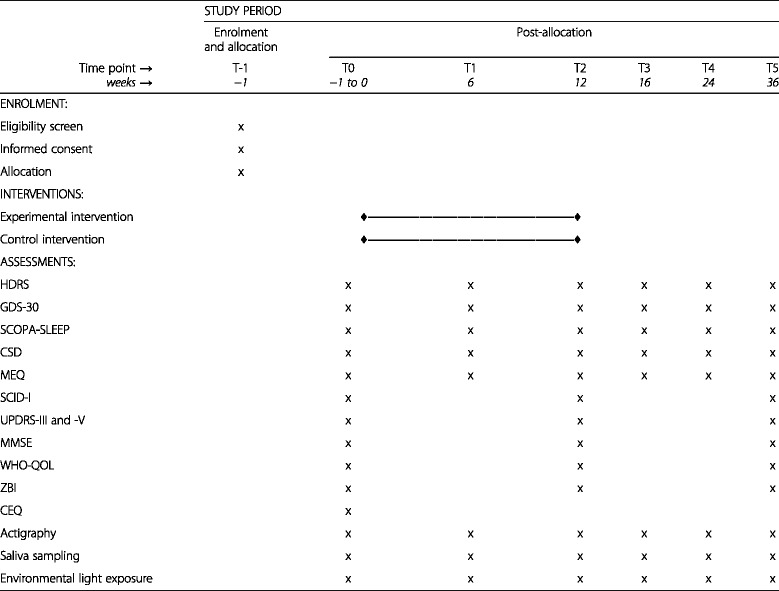
Overview of study procedures

*HDRS* Hamilton Depression Rating Scale, *GDS-30* 30-item Geriatric Depression Scale, *SCOPA-SLEEP* Scales for Outcomes in Parkinson’s Disease-Sleep, *CSD* Consensus Sleep Diary, *MEQ* Morningness-Eveningness Questionnaire, *SCID-I* Structured Clinical Interview for DSM-IV Axis I Disorders, *UPDRS* Unified Parkinson’s Disease Rating Scale, *MMSE* Mini Mental State Examination, *WHO-QOL-BREF* World Health Organization Quality of Life assessment, abbreviated version; *ZBI* Zarit Burden Interview, *CEQ* Credibility/Expectancy Questionnaire

After (self-)referral, subjects are sent an information package about the study. If still interested in participation after reading the additional information, prospective participants are screened by telephone by the first assessor (SR, psychiatry resident). When eligible, subjects are invited for an appointment with the first assessor. During this visit, subjects can ask any remaining questions. After providing written informed consent, subjects are randomized to the experimental or control condition by a research coordinator (CV), and baseline clinical assessments are performed. At the end of the visit, subjects are asked to perform actigraphy, keep a sleep diary and wear a light sensor at home for the upcoming seven days. After this week, all baseline assessments are completed and a BLT or control device is installed at the subject’s home by the study coordinator or a trained research assistant. After a demonstration of the device, the three-month treatment phase starts. Clinical assessments are repeated halfway (T1) and at the end of treatment (T2). After completing treatment, subjects enter a six-month follow-up phase. Subsequent interventions are allowed within the follow-up phase, since we consider it unethical to withhold treatment from patients with remaining depressive symptoms. Follow-up assessments take place one (T3), three (T4) and six months (T5) after completing treatment. Prior to each follow-up assessment, subjects receive a package containing questionnaires, a sleep diary, actigraphy watch, light sensor and cotton dental rolls for saliva sampling, to perform these measures at home in the week prior to the assessment visit.

### Randomization and blinding

To maintain blinding of the assessors, the research coordinator (CV) performs block randomization. Blocks are generated per season to rule out seasonal influences on the treatment effect. Both subjects and assessors are blinded for the treatment condition. To prevent unblinding, subjects are asked not to share any details of their treatment with their assessor. When patients do reveal their condition, the assessor is immediately replaced by the blinded project’s principle investigator (OvdH, psychiatrist).

### Participants

Participants for this study are recruited throughout the Netherlands by various strategies, including referral by a neurologist, psychiatrist or other medical professional, recruitment through media of various associations involved with PD, and advertisement in a Dutch national newspaper.

Patients are eligible for inclusion when they are diagnosed with idiopathic PD by a neurologist. In addition, they are required to meet criteria of a major depressive disorder as classified by the DSM-IV [45]. Patients with a bipolar disorder are excluded due to the risk of inducing (hypo)mania with BLT [38]. We also exclude subjects with a current psychosis, due to the potential influence on our assessments and the risk of noncompliance. Finally, we exclude patients with a (relative) contraindication for BLT, such as a disorder associated with photosensitization reactions to light, e.g. porphyria, macular degeneration, retinal dystrophy, or lupus erythematosus, or when they have used pharmacological agents with a photosensitizing effect within the past four weeks [20]. Because changes in the use of antiparkinsonian or psychiatric medication might influence assessments, participants have to be on a stable dose of medication for at least four weeks before inclusion. Moreover, subjects and their physicians are asked not to change the medication regime during the treatment phase of the trial, unless this is deemed medically necessary.

### Intervention

The experimental condition consists of BLT with a Brazil® Lightbox (Lumie, Cambridge, United Kingdom). This BLT device emits white, broad spectrum light. During treatment sessions, subjects are instructed to take place in front of the device at a distance of 30 cm, at which the intensity of the light is 10,000 Lux. To create a credible control condition, neutral density filters (Lee Filters, type 209.03ND, Hampshire, United Kingdom) are installed in the light device, so that the experimental and control device have an identical appearance when switched off. These filters reduce transmission uniformly, giving the light a dim white appearance. Subjects are instructed to position themselves at a distance of 40 cm from the control device, at which the light intensity is approximately 200 Lux. At this light intensity, which is lower than in a dimly lit room, no substantial effect on circadian rhythmicity is expected [46].

Subjects in both conditions follow the treatment at home for a period of three months. Subjects use the device both in the morning and the evening. Treatment duration per session is 30 min, which is sufficient to entrain the SCN at an intensity of 10,000 Lux [20]. Timing of the morning treatment session is based on the score on the MEQ [19, 41].

Since evening BLT should be administered at least 90 min prior to bedtime [20], the evening session takes place 9. Five hours prior to the morning BLT, in order to allow eight hours of nighttime sleep. To increase user convenience and compliance, the device is attached to a timer, so that the device switches on automatically at the times set for treatment.

### Outcomes

#### Primary outcome measure

The primary outcome measure of this study is the change in depressive symptoms as regards to baseline, as measured with the 17-item version of the Hamilton Depression Rating Scale (HDRS) [47], from baseline (T0) to the end of treatment (T2).

#### Secondary outcome measures

Our secondary outcome measures are (1) alternative depression measures, (2) subjective and objective quality of sleep, and (3) circadian rhythmicity. Since we are mainly interested in the direct and long-term treatment effects, all outcome measures described below concern the difference between the treatment groups in the change in outcome measures as regards to the baseline value between i) baseline and the end of treatment (T0 to T2) and ii) baseline and the end of follow-up (T0 to T5).

1. Additional depression measures:
  The change in HDRS-score between T0 and T5 is considered as a secondary outcome measure. As an alternative measure for depression, we use the Geriatric Depression Scale (GDS-30) [48], a self-report instrument. The GDS-30 does not contain items regarding somatic symptoms of depression, and might therefore have a higher diagnostic specificity in PD [48]. As a third depression measure, we assess the proportion of subjects in each treatment group that achieves complete remission at T2 and T5, i.e. fulfilling DSM-IV criteria of a ‘depressive disorder in full remission’, as determined with the Structured Clinical Interview for DSM-IV Axis I disorders (SCID-I) [49].
2. Sleep:
  - Subjective experience of sleep is measured with the Scales for Outcomes in Parkinson’s Disease-Sleep (SCOPA-SLEEP) [50], a validated self-report instrument that contains two subscales: one rating nighttime sleep disturbances and one on excessive daytime sleepiness. Both subscale scores are used as a secondary outcome measure.
  - Sleep patterns are objectified by actigraphy, which is considered to be a valid alternative for polysomnography in the quantitative evaluation of sleep in PD patients [51]. Subjects are asked to wear an actigraphy device (GENEActiv Sleep; GENEActiv, Huntingdon, United Kingdom) on their non-dominant wrist continuously for seven consecutive days. Sleep parameters are calculated in two steps. First, accelerometry data is preprocessed with GENEActive PC software version 2.2, using reports of time of attempted sleep and time of final awakening from the Consensus Sleep Diary (CSD) [52]. Second, the raw GENEActive accelerometry data are converted into Actiwatch counts, as described by Te Lindert et al. (2013), to calculate sleep parameters. The sleep parameters of main interest are total sleep time and sleep fragmentation, i.e. the number of wake bouts per night.
3. Circadian rhythmicity:
  In this study, we use salivary concentrations of melatonin and cortisol as markers for the circadian rhythm. At each time point in the study, subjects are instructed to collect saliva at home during a single day, using cotton dental rolls (Salivette®, Sarstedt, Numbrech, Germany). The first saliva sample is taken immediately after waking up, followed by three more samples at an interval of 30 min. In the evening, subjects start hourly collection of samples four hours prior to intended bedtime. Samples are preserved in the refrigerator by the patient, and collected by the researchers one or two days later. They are centrifuged and stored at −80 °C until all samples of one subject that are collected throughout the study can be analyzed simultaneously. The cortisol concentration in the saliva samples is determined by on-line solid-phase extraction liquid chromatography tandem mass spectrometry (XLS-MS/MS), which is preferred over immunoassays to prevent cross-reactivity with other corticosteroids [53]. We will calculate the area under the curve with respect to the ground (AUC_G_) and the area under the curve with respect to the index of salivary cortisol change over time (AUC_I_), as described by Pruessner et al. (2003) [54]. The AUC_G_ is an estimate of the total cortisol secretion throughout the day, whereas the AUC_I_ is a measure of the cortisol awakening response. The AUC_G_ and AUC_I_ of the salivary cortisol concentrations of the morning samples are considered as a secondary outcome measure. We will also report the cortisol concentration in the first saliva sample taken as a measure of the endpoint of pre-awakening increase in cortisol [17]. The melatonin concentrations are assessed in the evening saliva samples using liquid chromatography tandem mass spectrometry (LS-MS/MS). We will calculate the DLMO with the ‘hockey-stick method’, as described by Danilenko et al. (2013) [55].

#### Assessments for exploratory purposes

For exploratory purposes, we assess the difference between the treatment groups in the absolute change in the following outcome measure from T0 to T2 and T0 to T5.

1. Subjective quality of sleep, rated in the CSD on a scale ranging from 0 (very poor) to 4 (very good).
2. Sleep efficiency as objectified with actigraphy. Sleep efficiency is defined as the percentage of time the subject spent asleep between onset of sleep and getting up in the morning.
3. Evening cortisol secretion, calculated as both the AUC_G_ and AUC_I._
4. Global cognitive function, assessed with the Mini Mental State Examination (MMSE) [56].
5. Presence of comorbid psychiatric disorders, as determined with the SCID-I.
6. Severity of motor symptoms, using section III of the Unified Parkinson’s Disease Rating Scale (UPDRS) [57]. The UPDRS-III is rated by the first assessor, who is trained by a movement disorder specialist (HB).
7. The chronotype, as determined with the MEQ.
8. Quality of life, as measured with the abbreviated version of the World Health Organization Quality of Life assessment (WHOQOL-BREF) [58].
9. Caregiver burden, as reported by the caregiver on the Zarit Burden Interview (ZBI) [59].

#### Covariates

In order to control for factors that might influence the association between treatment and the outcome measures, we collect the following additional data:

1. Medication use:
  Current medication use is reviewed at each assessment. Since dopaminergic agents can have a positive influence on mood in PD patients with depressive symptoms [60], we will control for the total dose of dopaminomimetics, which is converted to a levodopa equivalent daily dose using the conversion rate described by Olde Dubbelink et al. (2013) [61]. Moreover, we will correct for the initiation or change in dosage of antidepressants or sleep medication.
2. Exposure to environmental light:
  We collect data on the exposure to environmental light using a light sensor (Actiwatch Light®, Cambridge Neurotechnology Ltd., Cambridge UK). Subjects are asked to continuously wear the sensor on the outer layer of their clothing for seven consecutive days. Raw data on light exposure is preprocessed with Actiwatch Activity & Sleep Analysis 5 software, after which total amount of light exposure during the day is calculated using custom-made in-house software.
3. Expectancy
  In a previous study on BLT, a positive correlation between expectations and treatment response was found [62]. In this study, we assess the expectancies of our subjects with the Credibility/Expectancy Questionnaire (CEQ) [63], after installation of the device at home.
4. Compliance:
  Compliance with treatment is assessed with an occupancy data logger (HOBO® occupancy/light logger) that is attached to the device. The logger detects the time and duration of presence of the participant in front of the device. Treatment session compliance is defined as exposure to the device for at least 70 % of the prescribed duration, i.e. 21 out of 30 min, within the set time frame. Total compliance is calculated as the proportion of sessions that a subject is compliant with therapy. We assess the effects of total compliance on the intervention effect, as well as compliance with morning and evening therapy separately.

#### Side effects and treatment satisfaction

Prior to the start of the trial, we made an overview of potential adverse effects that subjects might experience during treatment, based on previous reports [41, 64]. One week after the start of treatment, subjects are contacted by telephone by the research assistant to inquire about these side effects. At assessment visit T1, patients are also screened for side effects. We will report the difference in prevalence of reported side effects between both groups.

After completing the treatment phase, all patients are asked to rate their appreciation of timing and duration of therapy sessions on a visual analogue scale (VAS). Moreover, they indicate on a VAS the extent to which they would like to continue treatment after the end of the trial. We will report average VAS scores as an indicator of treatment satisfaction per group.

### Statistical analysis

#### Sample size calculation

Prior to the start of the study, we calculated the necessary sample size for a sufficient power at the end of treatment (T2) in a mixed models analysis. We aimed to have sufficient sensitivity for a minimal standardized effect size of Cohen’s d = 0.6, with an estimated standard deviation of 1.0 and intra-subject correlation of assessments of *ρ* = 0.6. For a statistical power of 80 % and a two-tailed significance level of *p* < 0.05, 35 subjects per treatment group were required. To correct for a maximum drop-out of 20 %, we would need 7 additional subjects per group, resulting in a total sample size of 84 subjects.

#### Efficacy analysis

Analyses will be performed on the Modified-Intent-To-Treat population, which consists of all subjects randomized in the trial who received at least one week of light treatment and provided at least one post-baseline assessment of the HDRS. As a sensitivity analysis, we will compare the baseline characteristics to the Intent-to-Treat population, i.e. all subjects who were randomized in the trial. To assess the effect of treatment duration, we will also assess the effects in the Per Protocol-population, i.e. all subjects who completed the three months treatment duration.

Demographics and clinical characteristics of both the BLT and control group at baseline will be presented with percentages or mean scores with standard deviations. Correlations between continuous variables at baseline will be calculated using Pearson’s correlation coefficients.

The effect of the intervention on the change from T0 to T2, and from T0 to T5, in outcome measures will be assessed using a linear mixed-effects model analysis with assessment visit as the lowest level, and patient as highest level. Time of assessment will be handled as a categorical variable. The effect of the intervention on the outcome measures over time will be modeled by an intervention by time interaction with two degrees of freedom. In all analyses, we will correct for baseline values of the outcome variable. In addition, we will assess whether adding the covariates to the regression model leads to a significant change of the regression coefficient of the condition effect of ≥10 %.

No imputation of missing values will be performed, since linear mixed models handle missing data by placing the data in long format, where the available data of each measurement is nested within persons.

## Discussion

Performing a placebo-controlled trial on the effects of BLT is challenging, since the appearance of the device or the characteristics of the emitted light can provide the subject with clues about the treatment condition. This can influence the subject’s expectancies of the treatment, which has been shown to impact on the outcomes of a study on BLT the past [62]. Deactivated negative ionizers, dim red light and light boxes with full-band filters were used as control light conditions for BLT in previous studies [37, 65]. In this trial, we also use a light device with full-band filter. However, the reduced intensity of the light could point out that the subjects in the control condition were not receiving BLT. Therefore, subjects’ expectancies are rated prior to the start of treatment, and participants are not informed about the exact details of the two light devices and their expected therapeutic effect. Although this might raise ethical questions, it is a valid way to guarantee subject blinding when studying a treatment that lacks a credible placebo condition. Therefore, this study did receive approval of the ethics committee. Moreover, the subjects in the control condition do receive some form of treatment. All subjects in our study are required to use the light device at fixed times in the morning and the evening, imposing a fixed sleep-wake structure on all participants. According to the ‘social zeitgeber’ hypothesis of Ehlers et al. (1988), a disruption of social rhythms can induce a depressive episode through disturbance of the circadian rhythm [66]. Restoring the circadian rhythm by re-establishing daily routines, including time of awakening and going to bed, might therefore have a positive influence on mood [66]. Since all participating subjects in our study achieve a more regular sleep-wake cycle, we feel that subjects in the control condition are not completely deprived of treatment. Moreover, a report by Zeiter et al. (2000) suggests that dim light of approximately 100 lux also has the ability to cause a shift in circadian rhythm [67]. Although the healthy volunteers participating in this study were exposed to the light for 6.5 h [67], we cannot rule out the possibility that 30 min of exposure to 200 lux of light will influence the circadian system of PD patients.

With our study design, we do not compare BLT to ‘pure’ placebo treatment, i.e. an ineffective control treatment. Rather, we compare structuring of the sleep-wake cycle through set treatment times in both conditions, with additional BLT in the experimental condition, and additional dim light in the control condition. In order to be able to assess the effects of BLT only, a future study on the effects of BLT on depression in PD would have two additional treatment arms, where BLT and control light are administered at random times. Moreover, more research is needed on the timing (morning or evening) and duration of BLT, as well as the influence of light intensity. However, we hypothesize that with the design of this study, we will still find a greater improvement on depression, insomnia and biomarkers of circadian rhythmicity in the subjects in our experimental condition as compared to our control subjects, due to the addition of BLT to the imposed sleep-wake structure.

## Abbreviations

AUC_G_: Area under the curve with respect to the ground
AUC_I_: Area under the curve with respect to the index of salivary cortisol change over time
BLT: Bright light therapy
CAR: Cortisol awakening response
CEQ: Credibility/Expectancy Questionnaire
CSD: Consensus Sleep Diary
DLMO: Dim-light melatonin onset
DSM-IV: Diagnostic Statistical Manual, fourth edition
GDS-30: 30-item Geriatric Depression Scale
HDRS: Hamilton Depression Rating Scale
LS-MS/MS: Liquid chromatography tandem mass spectrometry
MDD: Major depressive disorder
MEQ: Morningness-Eveningness Questionnaire
MMSE: Mini Mental State Examination
PD: Parkinson’s disease
RCT: Randomized controlled trial
SCID-I: Structured Clinical Interview for DSM-IV Axis I Disorders
SCN: Suprachiasmatic nucleus
SCOPA-SLEEP: Scales for Outcomes in Parkinson’s Disease-Sleep
UPDRS: Unified Parkinson’s Disease Rating Scale
WHO-QOL-BREF: World Health Organization Quality of Life assessment, abbreviated version
XLS-MS/MS: On-line solid-phase extraction liquid chromatography tandem mass spectrometry
ZBI: Zarit Burden Interview
